# Unlocking the complete blood count as a risk stratification tool for breast cancer using machine learning: a large scale retrospective study

**DOI:** 10.1038/s41598-024-61215-y

**Published:** 2024-05-12

**Authors:** Daniella Castro Araujo, Bruno Aragão Rocha, Karina Braga Gomes, Daniel Noce da Silva, Vinicius Moura Ribeiro, Marco Aurelio Kohara, Fernanda Tostes Marana, Renata Andrade Bitar, Adriano Alonso Veloso, Maria Carolina Pintao, Flavia Helena da Silva, Celso Ferraz Viana, Pedro Henrique Araújo de Souza, Ismael Dale Cotrim Guerreiro da Silva

**Affiliations:** 1 Huna, São Paulo, Brazil; 2 Grupo Fleury, São Paulo, Brazil; 3 Departamento de Análises Clínicas e Toxicológicas, Faculdade de Farmácia, Universidade Federal de Minas Gerais/UFMG, Campus Belo Horizonte, Minas Gerais, Brazil; 4 Departamento de Ciências da Computação, Instituto de Ciências Exatas, Universidade Federal de Minas Gerais/UFMG, Campus Belo Horizonte, Minas Gerais, Brazil; 5 Department of Oncology Clinical Research, Instituto Nacional de Câncer (INCA), Rio de Janeiro, Brazil; 6 Department of Gynecology, Escola Paulista de Medicina, Federal University of São Paulo, São Paulo, Brazil

**Keywords:** Breast cancer, Screening, Machine learning, Risk stratification, Routine blood tests, CBC, NLR, RBC, CBC-ratios, Hemogram, Risk factors, Diagnostic markers, Breast cancer

## Abstract

Optimizing early breast cancer (BC) detection requires effective risk assessment tools. This retrospective study from Brazil showcases the efficacy of machine learning in discerning complex patterns within routine blood tests, presenting a globally accessible and cost-effective approach for risk evaluation. We analyzed complete blood count (CBC) tests from 396,848 women aged 40–70, who underwent breast imaging or biopsies within six months after their CBC test. Of these, 2861 (0.72%) were identified as cases: 1882 with BC confirmed by anatomopathological tests, and 979 with highly suspicious imaging (BI-RADS 5). The remaining 393,987 participants (99.28%), with BI-RADS 1 or 2 results, were classified as controls. The database was divided into modeling (including training and validation) and testing sets based on diagnostic certainty. The testing set comprised cases confirmed by anatomopathology and controls cancer-free for 4.5–6.5 years post-CBC. Our ridge regression model, incorporating neutrophil–lymphocyte ratio, red blood cells, and age, achieved an AUC of 0.64 (95% CI 0.64–0.65). We also demonstrate that these results are slightly better than those from a boosting machine learning model, LightGBM, plus having the benefit of being fully interpretable. Using the probabilistic output from this model, we divided the study population into four risk groups: high, moderate, average, and low risk, which obtained relative ratios of BC of 1.99, 1.32, 1.02, and 0.42, respectively. The aim of this stratification was to streamline prioritization, potentially improving the early detection of breast cancer, particularly in resource-limited environments. As a risk stratification tool, this model offers the potential for personalized breast cancer screening by prioritizing women based on their individual risk, thereby indicating a shift from a broad population strategy.

## Introduction

Mammography screening is highly recommended for early-stage breast cancer (BC) detection and reducing mortality^1^. While the American Cancer Society (ACS) advises age-based screening guidelines, this approach may not consider individual risk factors^2^. Additionally, low- and middle-income countries often struggle to provide serial screening^1^. Therefore, adopting a reliable risk stratification tool to prioritize higher-risk women could optimize resource allocation by transitioning from population-based to individual screening^3^.

Several BC risk stratification tools have been proposed, with the Tyrer-Cuzick (TC) model^4^ being the most widely adopted and recommended by the ACS guidelines. This model leverages a composite of family history, demographic details, reproductive health insights, anthropometric data, and breast density to predict the risk of BC. However, despite its comprehensive approach, the TC model's accuracy remains limited. Furthermore, the specific data it requires might not be readily available in standard medical records, thereby necessitating an intricate and specialized data collection process. Recently, a deep learning model based on convolutional neural networks called MIRAI was introduced as a mammography-based risk assessment tool that predicts BC within five years, achieving a mean AUC of 0.78 in a multicenter evaluation^5^. Despite its effectiveness, the applicability of the MIRAI model can be limited in low-income countries due to a scarcity of mammograms, frequently resulting in the unavailability of necessary mammographic data for comprehensive risk assessment.

Liquid biopsy, a promising technology for early cancer detection, detects circulating tumor-associated cells^6^ using blood tests but remains expensive and less accessible. The prospect of cancer detection through a simple blood draw has always been appealing, especially for communities with limited medical access; yet, cost is a key factor for a genuine impact on public health. Consequently, affordable and globally accessible routine blood tests emerge as a timely solution, despite their general nonspecificity for diagnosis. Numerous studies have investigated the relationship between routine blood markers and BC, finding promising associations with complete blood count (CBC) parameters^7–9^.

Machine learning (ML) has been widely used to identify complex patterns in blood exams, enabling the combination of various markers to transform nonspecific tests into precise ones for multiple diseases. Here, we propose combining routine blood makers into a powerful AI-based panel to assess the risk of BC within six months of diagnosis. To our knowledge, this is the first study to utilize ML techniques to assess non-linear relationships between routine blood count test CBC markers for BC risk assessment.

## Methods

### Study design and patients

This retrospective study was approved by the Fleury Group's Research Ethics Committee (CEP-FG) (CAAE: 60984722.4.0000.5474), which is duly qualified by the National Research Ethics Committee (CONEP) of the National Health Council of Brazil. By decision of the CEP-FG, the requirement for informed consent was waived because of the retrospective and anonymized nature of the data. All research was carried out in compliance with the Brazilian legislation, General Data Protection Law** (**Lei Geral de Proteção dos Dados—LGPD), and the guidelines of the Declaration of Helsinki.

**Figure 1:**
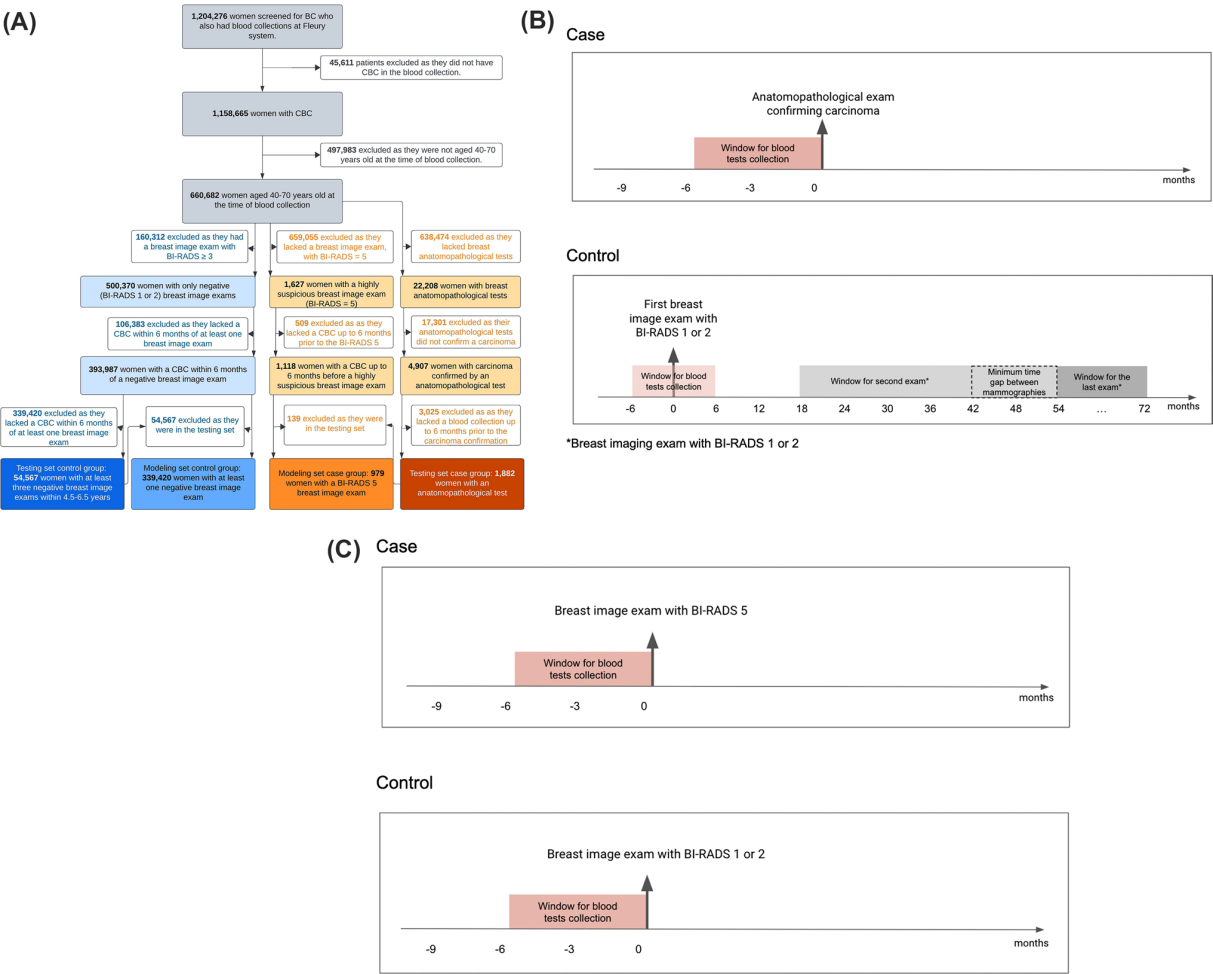
Schematic depiction of study design. (**A**) Flow diagram of the study population at the Fleury Clinical Labs that met all study inclusion criteria. (**B**) Time windows for blood exam collection for the testing dataset. (**C**) Time windows for blood exam collection for the modeling dataset.

We collected CBC test results from 396,848 women aged 40–70, screened for breast cancer (BC) between January 2004 and August 2022 across 309 Fleury clinical laboratory units in eight Brazilian states. Figure 1A shows the schematic diagram of the study population. Appendix P1 shows the geographic distribution of the states where the blood exams were collected.

The labeling of CBCs was performed by mining carcinoma confirmations from anatomopathological reports or the breast imaging-reporting and data system (BI-RADS) categories from breast imaging reports (mammograms, ultrasounds, or magnetic resonance imaging).

The case group comprised women diagnosed with BC, confirmed by an anatomopathological exam, or highly suspected of it (BI-RADS 5, indicating a cancer likelihood of > 95%), with a blood test collected up to six months before their breast biopsy or imaging exam. The control group included women with negative imaging exams (BI-RADS 1 or 2). Due to the varying levels of diagnostic certainty, we partitioned the database into two main segments: the test set, for the highest certainty, and the modeling set, which combines training and validation sets. These are explained below, and are summarized in Appendix P2.

- Test Set: This set included 1,882 cases anatomopathologically confirmed with a CBC conducted up to six months before breast biopsy. Controls were women who remained cancer-free for at least 4.5–6.5 years, encompassing women with at least three negative (BI-RADS 1 or 2) breast image exams within this period, spaced apart by at least 18 months. Their CBCs were collected in a 6-month window centered on the baseline image exam, totaling 54,567 CBCs, one per woman. For individuals with multiple tests, only the first CBC test was considered. Dataset construction time windows for the test set are illustrated in Fig. 1B.
- Modeling Set: This set included 979 BI-RADS 5 cases, each with one blood analysis conducted up to six months before the breast image exam. Controls included 339,420 women with one negative (BI-RADS 1 or 2) result, each represented by a CBC conducted up to six months before the breast image exam. The modeling set was further segmented into training (80%) and validation (20%) phases. Dataset construction time windows for the modeling set are detailed in Fig. 1C.

#### Testing set case group characterization

##### Carcinoma subtypes

We mined the carcinoma subtypes from the anatomopathological reports. Of the 1882 women with BC, 5% did not have information about the invasion status of BC in the reports. Of the remaining 1794, 1357 (76%) were invasive and 437 (24%) were in situ.

Of the 1357 women with invasive carcinoma, histologic subtypes could not be determined for 20% of the cases. Of the remaining 1080, 713 (66%) were ductal, 313 28%) lobular, and 54 (5%) of other subtypes.

Of the 437 women with in situ carcinoma, 50% had necrosis. Histologic subtypes could not be determined for 11% of the cases. Of the 388 remainings, 362 (93%) were ductal and 26 (7%) lobular. Regarding the grades of the 362 ductal carcinomas in situ, 27% did not have information about the grade. Of the remaining 263, 82 (31%) were considered low-grade, 85 (32%) intermediary grade, and 96 (36%) high-grade^10^.

##### Molecular subtypes

Among the 1357 women with invasive breast cancer, 774 also underwent an immunohistochemical (IHC) examination, where estrogen, progesterone, and HER2 receptors were assessed. Employing IHC surrogates to identify molecular subtypes, tumors were classified as:

- Luminal, if estrogen receptor-positive and/or progesterone receptor-positive, and HER2-negative: 532 (69%)
- Luminal with HER2, if estrogen receptor-positive and/or progesterone receptor-positive, and HER2-positive: 170 (22%)
- HER2-enriched, if estrogen receptor-negative, progesterone receptor-negative, and HER2-positive: 29 (5%)
- Triple-negative, if estrogen receptor-negative, progesterone receptor-negative, and HER2-negative: 43 (4%)

##### T staging

Using the main lesion's description from the imaging tests, the invasive tumors were classified into three T staging groups, taking into consideration the classification of tumor size^11^. T staging could not be determined for 11% of the invasive carcinomas. Of the remaining 1209 cases, T stagings were estimated as:

- T1, if tumor size was smaller or equal than 2 cm: 868 (72%)
- T2, if tumor size was bigger than 2 and smaller or equal than 5 cm: 282 (23%)
- T3, if tumor size was bigger than 5 cm: 59 (5%)

**Figure 2:**
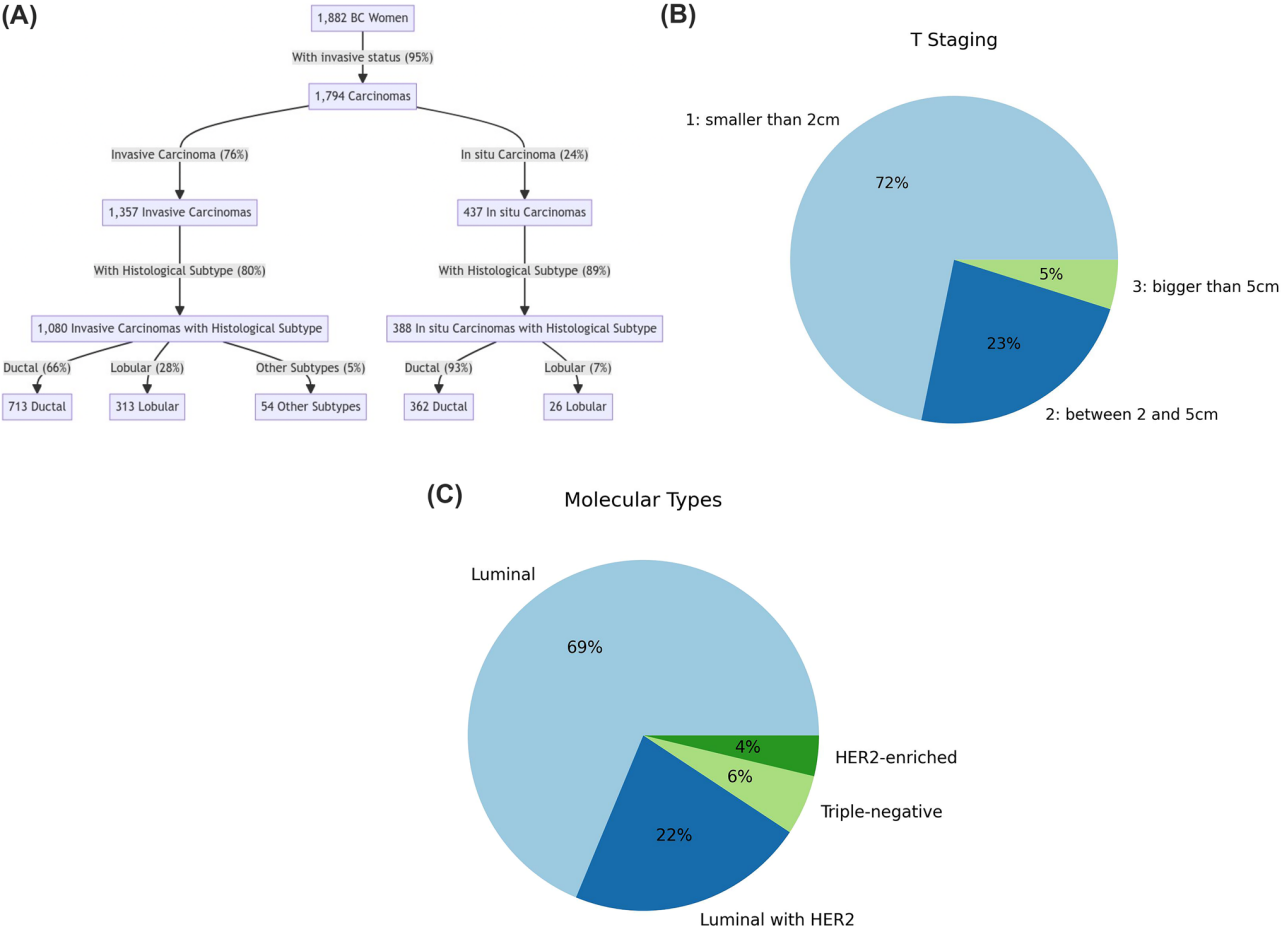
Overview of case group characteristics. (**A**) Flowchart detailing the status of invasive carcinoma and its histological subtypes. (**B**) Pie chart illustrating the distribution of molecular subtypes among the invasive carcinomas with immunohistochemical examinations (57% of the cases); (**C**) Pie chart depicting the T staging of invasive carcinomas of which the tumour size could be determined (89% of the cases).

Figure 2 provides a visual overview of the case group characteristics.

### Materials

CBC measurements were obtained from EDTA-K3 peripheral blood samples analyzed by the Automated Hematology Analyzer XT or XN series from Sysmex (Sysmex Corporation, Kobe, Japan). Red blood cells (RBC) and platelets were counted and sized through direct current impedance, while hematocrit and hemoglobin levels were determined using pulse height and sodium lauryl sulfate spectrophotometry, respectively.

### AI-based models

Our main goal was to use supervised learning to classify blood tests collected from BC patients up to six months before the diagnosis *versus* those from control patients.

Models were developed using two different machine learning models: ridge regression^12^ and LightGBM^13^. Ridge Regression is highly interpretable, enhancing linear regression by incorporating an L2 regularization term. This term penalizes large coefficients to prevent overfitting and address multicollinearity among predictors. On the other hand, LightGBM is a gradient-boosting algorithm known for its efficiency and effectiveness. It builds models sequentially, correcting errors from previous trees, and is particularly suited for handling large datasets, providing high accuracy with reduced training time. Their performance was evaluated through the Area Under the Curve (AUC) metric. For each experiment, we provide the 95% confidence intervals of the AUC, ensuring statistical significance. This was accomplished by conducting 1000 bootstrap resamplings with replacement of the training dataset.

Initially, our dataset comprised 13 CBC biomarkers, along with age, resulting in a total of 14 features. Subsequently, we calculated seven CBC-derived ratios, named neutrophils-lymphocyte ratio (NLR), derived neutrophils-lymphocyte ratio (dNLR), lymphocyte-monocyte ratio (LMR), platelet-lymphocyte ratio (PLR), systemic immune-inflammation index (SII), systemic inflammation response index (SIRI), and aggregate index of systemic inflammation (AISI). The formulas for each are detailed in Appendix P3. Utilizing all 21 features, we proceeded to make an initial prediction for both algorithms.

Given that our dataset was complete with no missing values, no imputation or missing data treatment methods were necessary. Features were normalized based on the training set min–max values to ensure uniformity in scale. To prevent data leakage, hyperparameter tuning and feature selection were conducted solely within the modeling dataset. The testing dataset was reserved strictly for final evaluation.

Following this, we implemented a feature selection method guided by a directed acyclic graph. This approach begins by identifying all potential models with a single feature, selecting the one with the highest AUC value. We then extend our search to models with two features, again choosing the one with the best AUC. This process of enumerating models and selecting the best-performing one based on AUC continues, with one additional feature being incorporated at each step. The procedure halts once the improvement in AUC no longer exceeds its standard deviation, ensuring a balance between model complexity and performance^14,15^.

Given the low prevalence of breast cancer (BC) in the general population, we observed a significant imbalance in the target variable within both the modeling and testing sets, at 0.29% and 3.3%, respectively. To address this imbalance, we applied higher weights to the cases in the training of both models.

For Lightgbm explainability analysis, we used the SHapley Additive exPlanations (SHAP)^16^ approach. SHAP evaluates feature importance by measuring the impact of omitting each feature on the model's decision. It produces SHAP values for every input, indicating each feature's significance in predictions.

After applying the best-performing model, we divided the population into four risk categories (high, moderate, average, and low) based on the model's probability outputs, following the methodology suggested by Michaels et al.^17^. We used the relative risk (RR) metric to measure the likelihood of breast cancer occurrence in each group relative to the population of this group. Appendix P4 presents a diagram outlining the methodology steps we employed.

## Results

### Statistical analysis

This retrospective study included CBCs from 396,848 women aged 40–70 years, of which 2861 (0.72%) were diagnosed or highly suspicious of BC up to 6 months after the blood collection. We did not investigate demographic or clinical data, such as ethnicity, as these fall out of laboratory records. Table 1 shows the linear descriptive statistics for all primary features and the CBC ratios for the full dataset. P-values were calculated using the Student’s t-test, and values ≤ 0.002 were considered significant (alpha was corrected from 0.05 to 0.001 using the Bonferroni test). Predictive AUCs were estimated by training a two-level decision tree on the training set and evaluating it on the test set. To estimate the 95% confidence interval, we performed 1000 bootstrap resamplings on the training set.

**Table 1:** Descriptive statistics of blood markers and age for both groups of the full dataset.

Exam	Feature	Control Mean ± STD	Case Mean ± STD	P value	Predictive AUC (95% CI)
	Age (years)	50.04 ± 8.56	53.92 ± 8.50	**0.000**	0.60 (0.58–0.61)
CBC	Eosinophils (/mm^3^)	173.62 ± 149.65	170.59 ± 140.29	0.281	0.50 (0.49–0.51)
	Hematocrit (%)	39.84 ± 2.96	40.02 ± 3.18	**0.002**	0.51 (0.50–0.52)
	Hemoglobin (g/dL)	13.23 ± 1.09	13.31 ± 1.15	**0.000**	0.50 (0.49–0.52)
	Leukocytes (/mm^3^)	6421.13 ± 1894.44	6344.95 ± 1889.42	0.032	0.50 (0.50–0.51)
	Lymphocytes (/mm^3^)	2079.67 ± 670.97	2000.46 ± 648.53	**0.000**	0.51 (0.50–0.51)
	MCH (pg)	29.47 ± 2.08	29.66 ± 2.05	**0.000**	0.50 (0.49–0.51)
	MCHC (%)	33.19 ± 1.04	33.25 ± 0.98	0.005	0.50 (0.49–0.50)
	MCV (fL)	88.75 ± 5.19	89.19 ± 5.21	**0.000**	0.50 (0.50–0.51)
	Monocytes (/mm^3^)	479.78 ± 155.22	480.73 ± 164.89	0.744	0.50 (0.50–0.51)
	Neutrophils (/mm^3^)	3650.25 ± 1468.55	3654.07 ± 1483.28	0.890	0.50 (0.49–0.50)
	Platelets (10^3^/mm^3^)	256.8 ± 57.2	261.9 ± 61.1	0.540	0.50 (0.49–0.52)
	RBC (10^6^/mm^3^)	4.50 ± 0.37	4.50 ± 0.40	0.831	0.51 (0.50–0.52)
	RDW (%)	13.33 ± 1.14	13.36 ± 1.14	0.067	0.48 (0.47–0.50)
CBC ratios	AISI (× 10^6^)	236.8 ± 196.7	252.3.0 ± 219.4	**0.000**	0.51 (0.50–0.52)
	dNLR	1.37 ± 0.57	1.42 ± 0.62	**0.000**	0.51 (0.50–0.52)
	LMR	4.60 ± 2.03	4.45 ± 1.64	**0.000**	0.51 (0.50–0.51)
	NLR	1.87 ± 0.90	1.97 ± 1.01	**0.000**	0.51 (0.50–0.52)
	PLR	136.11 ± 47.12	143.06 ± 53.81	**0.000**	0.52 (0.50–0.53)
	SII (× 10^3^)	481.4 ± 262.7	509.2 ± 287.6	**0.000**	0.51 (0.50–0.52)
	SIRI	921.53 ± 725.26	979.91 ± 836.79	**0.000**	0.51 (0.50–0.51)

P-values ≤ 0.002 are in bold.

MCH = mean corpuscular hemoglobin; MCHC = mean corpuscular hemoglobin concentration; MCV = mean corpuscular volume; MPV = mean platelet volume; RBC = red blood count; RDW = red cell distribution width; AISI = aggregate index of systemic inflammation; dNLR = derived NLR; LMR = lymphocytes-to-monocytes ratio; NLR = neutrophils-to-lymphocytes ratio; PLR = platelets-to-lymphocytes ratio; SII = systemic immune-inflammation index; SIRI = systemic inflammation response index.

In addition to age, the biomarkers hemoglobin, hematocrit, mean corpuscular hemoglobin (MCH), and mean corpuscular volume (MCV) were significantly higher in women with BC, whereas lymphocyte levels were significantly lower compared to the control group. All CBC-derived ratios were significant and elevated in BC cases, except for the LMR, which was lower. Age alone was the sole feature capable of distinguishing between the two groups, yielding a predictive AUC of 0.60 (95% CI 0.58–0.61).

### Models' performance and selected features

The selected features for both models were the biomarkers neutrophils-lymphocyte ratio (NLR), red blood cells (RBC), plus age. The AUC performance of the developed models is reported in Table 2 for the validation and testing sets.

**Table 2:** AUC (95% CI) for both models and both feature scenarios.

Model	Features	Validation (95% CI)	Testing (95% CI)
LightGBM	All features	0.64 (0.61–0.66)	0.62 (0.61–0.64)
	NLR, RBC and age	0.65 (0.63–0.67)	0.63 (0.62–0.64)
Ridge regression	All features	0.65 (0.64–0.66)	0.63 (0.62–0.64)
	NLR, RBC and age	0.66 (0.65–0.66)	0.64 (0.64–0.65)

The LightGBM algorithm performs slightly worse than the ridge regression on both datasets. Besides, it is less stable. Further, an important benefit of ridge regression is its full interpretability, making it a transparent choice for analysis. Given these factors, and with an AUC of 0.64 (95% CI 0.64–0.65) on the testing set, ridge regression has been selected as the best model.

### Model explainability analysis

**Figure 3:**
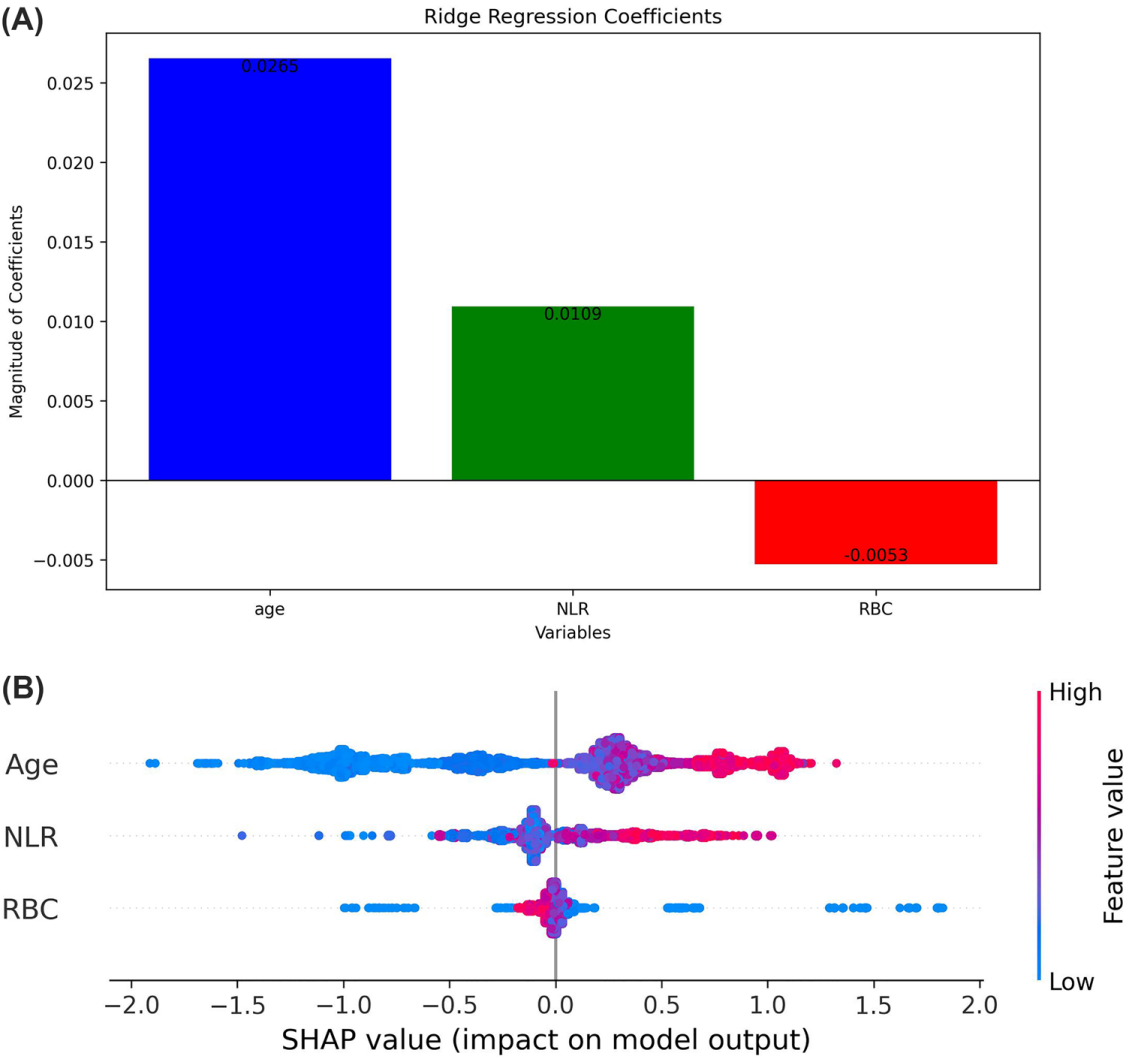
Explainability analysis. (**A**) Bar graph displaying Ridge Regression coefficients. (**B**) SHAP summary plot for the LightGBM model, showing the effect of each feature on BC risk prediction. Features are depicted in the order of importance. Pink dots are associated with women for which the corresponding feature shows a relatively higher value and blue dots with the opposite. Positions further to the right signify a greater predicted BC risk. NLR = neutrophils-to-lymphocytes ratio; RBC = red blood cells.

In this section, we analyzed the influence of individual features on the decision-making process. Leveraging the fully interpretable nature of ridge regression, we visually represent its coefficients through a bar graph (Fig. 3A). For the LightGBM model, the SHAP Summary Plot (Fig. 3B) displays features in order of importance, with pink, purple, and blue dots representing relatively higher, intermediary, and lower values, respectively. A vertical line separates women based on the model's probability, where positions further to the right signify a greater predicted BC risk. Notably, while features are evaluated individually, their importance considers non-obvious interactions among all features within the model.

Figure 3A illustrates the ridge regression coefficients, arranged by their importance—namely, age, NLR, and RBC. Figure 3B similarly presents the LightGBM model's features, in a matching order of significance. In the ridge model, an elevation in age and NLR is linked to an increased BC risk, whereas a rise in RBC is associated with a reduced risk. The LightGBM model demonstrates a similar trend: for age and NLR, there's a predominance of blue dots on the left, shifting to purple and then pink towards the right, indicating that higher values of these features correlate with a heightened BC risk. Conversely, RBC exhibits an opposite pattern, suggesting that lower levels of this marker are linked to an increased BC risk. This comparison highlights the congruence in decision-making processes between the two models, illustrating that their determinations regarding risk factors are consistent with one another.

### Risk stratification

To simulate a real-world application of this risk stratification tool, we suggest utilizing the ridge model's output probabilities to categorize the population into different risk levels, thereby facilitating the creation of prioritization waves to optimize mammographic screening resources.

We have modified the framework proposed by Michaels et al.^17^, which categorizes patients into three risk groups based on a review of risk factors: high (for those with a 20% or greater lifetime risk), moderate (for those with a 15–20% lifetime risk), and average (for a 15% or lower lifetime risk). In our tailored version of their framework, we've computed the ratio of lifetime risks by comparing each risk group against the baseline established by the average risk category. As a result, the high, moderate, and average groups are characterized by Relative Risks (RR) of greater than 1.3, between 1.0 and 1.3, and less than 1.0, respectively. We also integrated a low risk segment for individuals exhibiting an RR beneath 0.8.

To categorize the population into the defined groups, we segmented the testing database into ten equal percentiles. Subsequently, we determined the ratio of BC cases to the total population within each percentile and further grouped them based on each Relative Risk (RR) value. Finally, we computed the average RR for each risk category. The direct equation for the ridge model application and respective thresholds used for this classification are detailed in Appendix P5.

The first priority group, classified as high risk, encompassed 10.0% of the total population but accounted for 19.8% of all diagnosed cancer cases, resulting in a Relative Risk (RR) of 1.99. The second group, named moderate risk, comprised 20.0% of the population and was associated with 26.5% of the cancer diagnoses, yielding an RR of 1.32. The third group, representing average risk, included 40.0% of the population while corresponding to 41.1% of the cancer cases, with an RR calculated at 1.02. Lastly, the fourth group, labeled as low risk, constituted 30.0% of the population but only 12.6% of the cancer cases, reflecting an RR of 0.42. These metrics are summarized in Appendix P6.

### Model analysis for different BC characteristics

**Figure 4:**
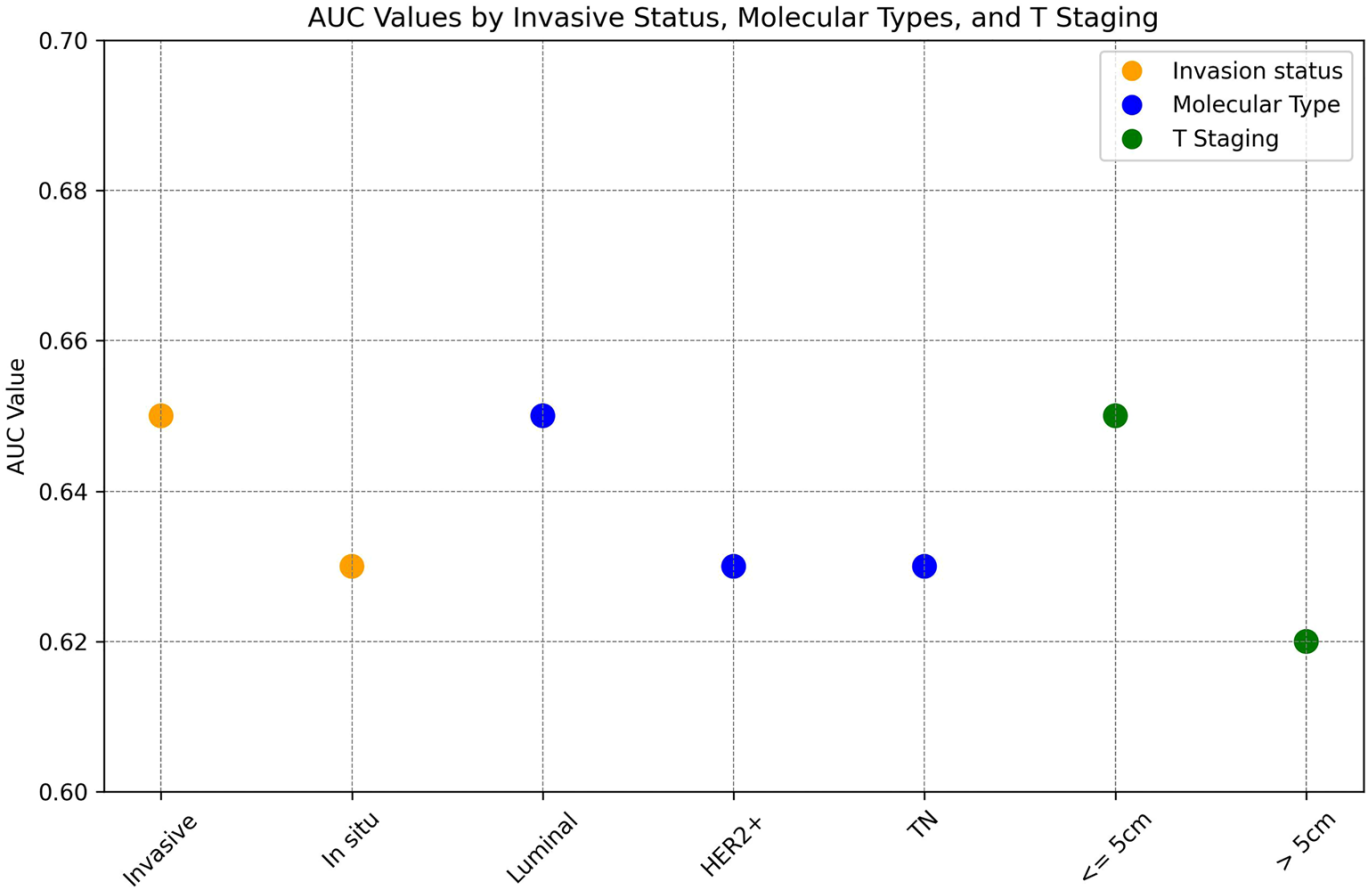
Model's Performance in different populations of the case group. HER2 = Luminal with HER2 or HER2-enriched; TN: Triple Negative.

To understand the ridge model's performance across different tumor characteristics, we have measured the AUC considering subsets of the testing case group while maintaining the full control group. Figure 4 plots the AUC values by case subset.

Regarding the BC invasion status, the model showed slightly improved performance for invasive tumors (AUC 0.6) compared to in situ tumors (AUC 0.63). In terms of molecular subtypes, the model was slightly more effective for luminal tumors (AUC 0.65) than for triple-negative and HER2+ tumors (AUC 0.63). Concerning T Staging, the model demonstrated marginally better results for smaller tumors than for larger ones (0.65 vs. 0.62).

## Discussion

The possibility of early cancer detection through a simple blood draw has always attracted attention, which presents an opportunity for low-resource countries to improve screening programs. Our study showed that routine laboratory biomarkers from CBC could be useful for early BC risk identification (within six months of the diagnosis), enabling further prioritization for screening. Since CBCs are affordable and often performed during routine health check-ups, our approach would not significantly elevate health costs. It simply leverages a widely-used test to provide invaluable insight into BC risk, resulting in a negligible increase in cost relative to the potential benefits of early detection.

Statistical analysis revealed that age, hematocrit, hemoglobin, MCH, MCV, AISI, dNLR, NLR, PLR, SII, and SIRI were significantly elevated in women with BC, whereas lymphocytes and the LMR were markedly lower. Given the statistical significance of all CBC-derived ratios, they present a valuable opportunity as cost-effective predictive biomarkers in BC. Furthermore, by harnessing AI, we developed a ridge regression model incorporating age, NLR, and RBC, achieving an AUC of 0.64 (95% CI 0.64–0.65), demonstrating superior performance over more complex models like LightGBM, while offering full interpretability.

To emulate the use of a risk stratification tool, we divided our study population into four risk groups: high, moderate, average, and low. This division was motivated by the necessity to effectively manage mammography queues, a challenge particularly relevant in countries like Brazil, where only 20% of women can access mammography via the public health system (SUS)^18^, a circumstance exacerbated by the COVID-19 pandemic. Given such resource scarcity or lengthy waiting times—a common situation in Brazil and many other countries—a risk stratification model for screening proves extremely beneficial. This stratification dictates which women should be screened first, starting with the high-risk group, followed by the moderate-risk group, and so forth, contingent on mammography resource availability. These groups were sorted based on the likelihood of BC occurrence in each group relative to the population of this group. Distinct risk profiles emerged, showing that the first to fourth groups had a BC probability of 1.99, 1.32, 1.02, and 0.42 times the typical risk. Hence, this model could serve as a tool for screening prioritization based on individual risk, enhancing the speed and efficiency of BC case identification, particularly in settings with limited resources. Importantly, the four-group categorization is a demonstrative application of the model's output. However, priority class configurations and allocations can be tailored to meet specific needs, such as accounting for demographic variations or adjusting to the screening capacities of various institutions.

While our dataset lacked specific indicators for identifying metastasis and lymph node status required for TNM staging, it is important to note that the BC study population in the testing set primarily consisted of early-stage carcinomas, as reflected by the fact that 24% of the identified carcinomas were in situ and 95% of the recorded invasive T stages were at T1-2. It is important to note that the modeling set cases did not undergo anatomopathological and immunohistochemical exams at Grupo Fleury, potentially differing in BC characteristics from the testing set. Despite this, the model maintained consistent performance across all BC subtypes. Our analysis revealed the model was slightly more effective for invasive carcinomas than in situ ones (AUC 0.65 vs. 0.63), indicating more distinct ML model patterns as the carcinoma advances. For T Staging, the model showed slightly improved accuracy for tumors smaller than or equal to 5 cm, with an AUC of 0.65 compared to 0.62 for larger tumors. However, the group with tumors larger than 5 cm was significantly smaller, consisting of only 59 individuals. Additionally, the model exhibited slightly higher performance for luminal subtypes compared to HER2 and triple-negative carcinomas, with an AUC of 0.65 versus 0.63.

The explanatory analysis of our model reveals that elevated levels of age and NLR and lower values of RBC may be indicative of an increased risk of BC. It is well known that advanced age is associated with the incidence of breast cancer due to several factors: (1) cumulative exposure^19^: as women age, they are exposed to various risk factors, mainly hormones, but also to other environmental carcinogens; (2) genetic mutations^20^: inherited and spontaneous mutations can accumulate in breast cells and lead to cancerous changes; (3) cellular aging^21^: a reduction in the efficiency of DNA damage repair can lead to a higher risk of cancer, as damaged or abnormal cells can multiply uncontrollably.

It has been proposed that high NLR, calculated as neutrophils/lymphocytes, may be indicative of an inflammation process^22^. In the context of BC, neutrophils might have a dual role: on one hand, they can suppress the immune response and foster tumor growth by inhibiting lymphocytes and T-cell activity^23^. On the other, they possess anti-cancer capabilities, killing tumor cells when activated in tissues^24^. The presence of high neutrophil levels in the blood thus may reflect their reduced activation in tissues where they could exert anticancer effects^25^. Transitioning to lymphocytes, these cells serve as essential components in the anti-tumor immune response, inducing cytotoxic death and inhibiting the proliferation and migration of tumor cells^26^. Thus, a decrease in lymphocyte count could indicate impairment of the immune response to tumor progression. Higher NLR levels have already been associated with an increased risk of BC^25^, with worse BC and solid tumours prognosis^22,27^ and also with an increased risk of other types of cancer^28,29^.

RBC plays a vital role in oxygen transport throughout the body. In the context of cancer, this relationship can be complex, and the exact role of erythrocytes in the pathogenesis of breast cancer is not yet fully understood. However, studies suggest that a chronic inflammatory state, which may be associated with a lower erythrocyte count, can promote the development and progression of cancer^30^.

### Limitations

Our study has strengths and limitations. First, the strengths include the fact that a total of 396,848 women coming from 309 laboratories spread in eight Brazilian states were included, with blood exams conducted using globally world-adopted equipment and largely standardized biochemical measurements in a College of American Pathologists (CAP) accredited institution. Several limitations, however, should be noted. Information about previous comorbidities and use of drugs was not available, which could bias our study and interfere with the analyzed blood tests. Additionally, the lack of access to comprehensive demographic and clinical information, including ethnicity, could influence our results. Another critical limitation is the absence of external validation to corroborate our findings across different populations and settings. Furthermore, we acknowledge that the thresholds provided for risk stratification, while based on our study population, may require adjustment for application to populations with different age distributions, or to accommodate varying screening capacities and health care protocols of different institutions. This acknowledges the need for a flexible approach to applying our model, underscoring that different thresholds may be more appropriate depending on specific population characteristics or institutional capabilities.

### Conclusion

In conclusion, by leveraging CBC biomarkers and age, which are routinely obtained from standard blood tests conducted annually, we developed an AI-based tool for BC risk stratification. This tool provides a potential pathway to a risk-based screening strategy, thereby generating a tangible impact on public health. While clinical decision-making often relies on cutoff points, ML models can perform better by detecting non-linear relationships between markers, thereby revealing the power of hidden patterns. Thus, the same routine CBC blood test could offer additional value by feeding data into our ML tool, with no need for new or additional tests. This tool could be integrated into various clinical settings, either through an application where a physician inputs blood marker data or as an API linked directly to a laboratory or health operator conducting a blood test, assigning each test a BC risk score. To support its clinical application, external validation with diverse populations is of paramount importance.

### Supplementary Information

Supplementary Information.

## Data Availability

Data used in this study are restricted due to confidentiality limitations. Requests should be directed to bruno.arocha@grupofleury.com.br and will undergo internal approval from Grupo Fleury.
