# Supplementary material for: Unlocking the complete blood count as a risk stratification tool for breast cancer using machine learning: a large scale retrospective study
Source: Sci Rep. 2024 May 12;14:10841. doi: 10.1038/s41598-024-61215-y (PMC11089041; doi:10.1038/s41598-024-61215-y)
Supplement: Supplementary file 1 — Supplementary Information. [file 41598_2024_61215_MOESM1_ESM.pdf]

Supplementary appendix

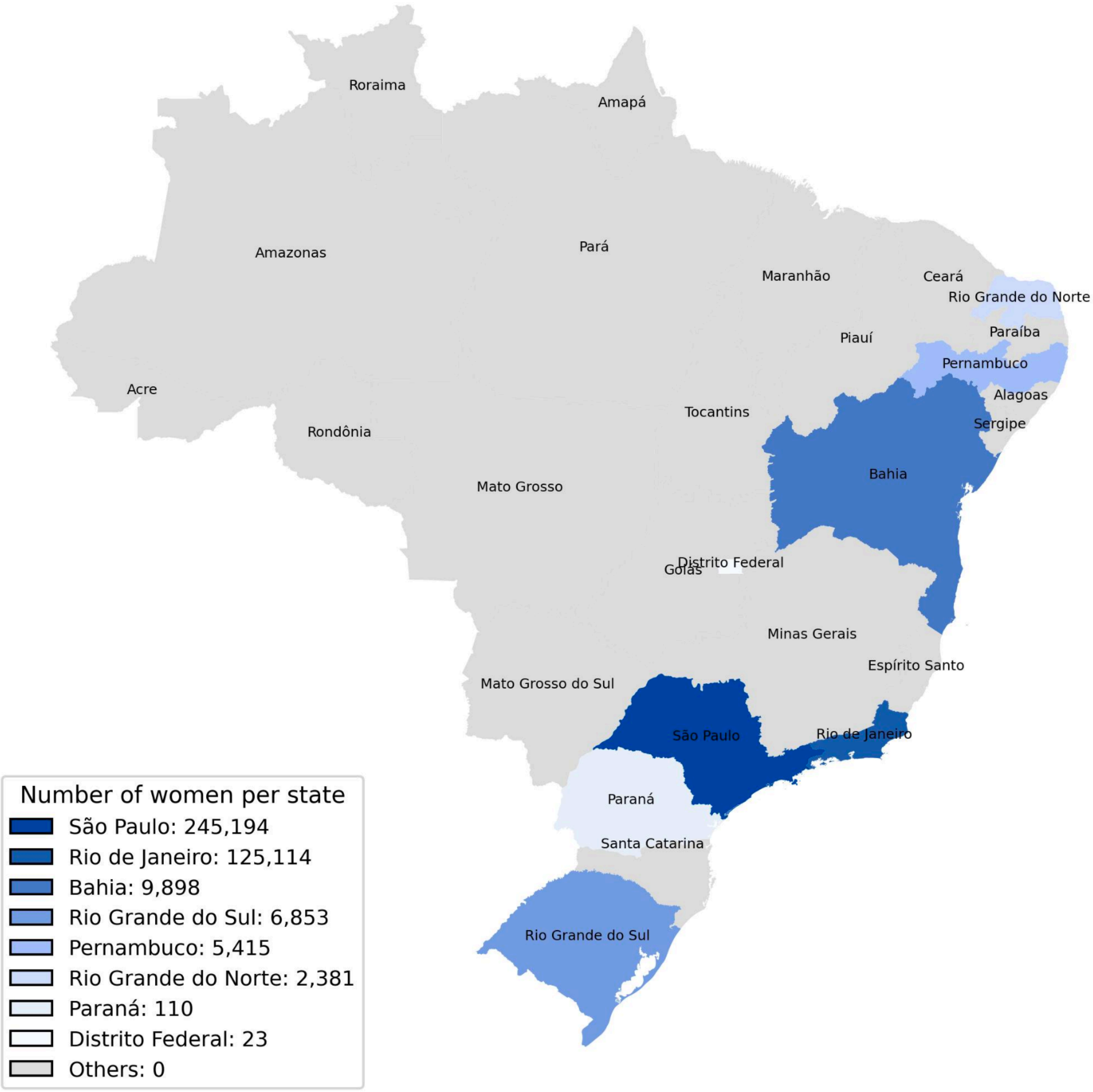

Appendix P1 - Map of Brazil, showing the states where the blood samples were collected. This map was generated using GeoPandas version 0.14.2 (Jan 4, 2024), an open-source Python library for geospatial data manipulation (https://geopandas.org). The geographical boundaries of Brazilian municipalities by state were sourced from the geodata-br repository (https://github.com/tbrugz/geodata-br), which provides Geojson files with municipal perimeters based on data from the Brazilian Institute of Geography and Statistics (IBGE) (https://ibge.gov.br/). The geodata-br repository is licensed under Creative Commons CC0 1.0 Universal (https://creativecommons.org/publicdomain/zero/1.0/).

Database	Breast Cancer (case)	Cancer free (control)	Total
**Modeling set (lower certainty)**	Women highly suspected of BC (>95%) by a breast image exam categorized as BI-RADS 5 (N = 979)	Women with one negative breast image exam categorized as BI-RADS 1 or 2 (N = 339,420)	340,399
**Testing set (higher certainty)**	Women diagnosed with BC, confirmed by an anatomopathological exam (N = 1,882)	Women who remained cancer-free for at least 4.5-6.5 years, with at least three negative breast image exams categorized as BI-RADS 1 or 2 within this period, spaced apart for at least 18 months (N = 54,567)	56,449
**Total**	2,861	393,987	396,848

Appendix P2 - Datasets labeling. Each woman is represented by a complete blood count (CBC)

Marker	Formula
NLR	$\frac{Neutrophils}{Lymphocytes}$
PLR	$\frac{Platelets}{Lymphocytes}$
MRL	$\frac{Lymphocytes}{Monocytes}$
dNLR	$\frac{Neutrophils}{Leukocytes-Neutrophils}$
SIRI	$Neutrophils \times \frac{Monocytes}{Lymphocytes}$
AISI	$Neutrophils \times Monocytes \times \frac{Platelets}{Lymphocytes}$
SII	$Platelets \times \frac{Neutrophils}{Lymphocytes}$

Appendix P3 - CBC derived ratios formulas. Abbreviations: AISI = aggregate index of systemic inflammation; dNLR = derived NLR; LMR = lymphocytes-to-monocytes ratio; NLR = neutrophils-to-lymphocytes ratio; PLR = platelets-to-lymphocytes ratio; SII = systemic immune-inflammation index; SIRI = systemic inflammation response index.

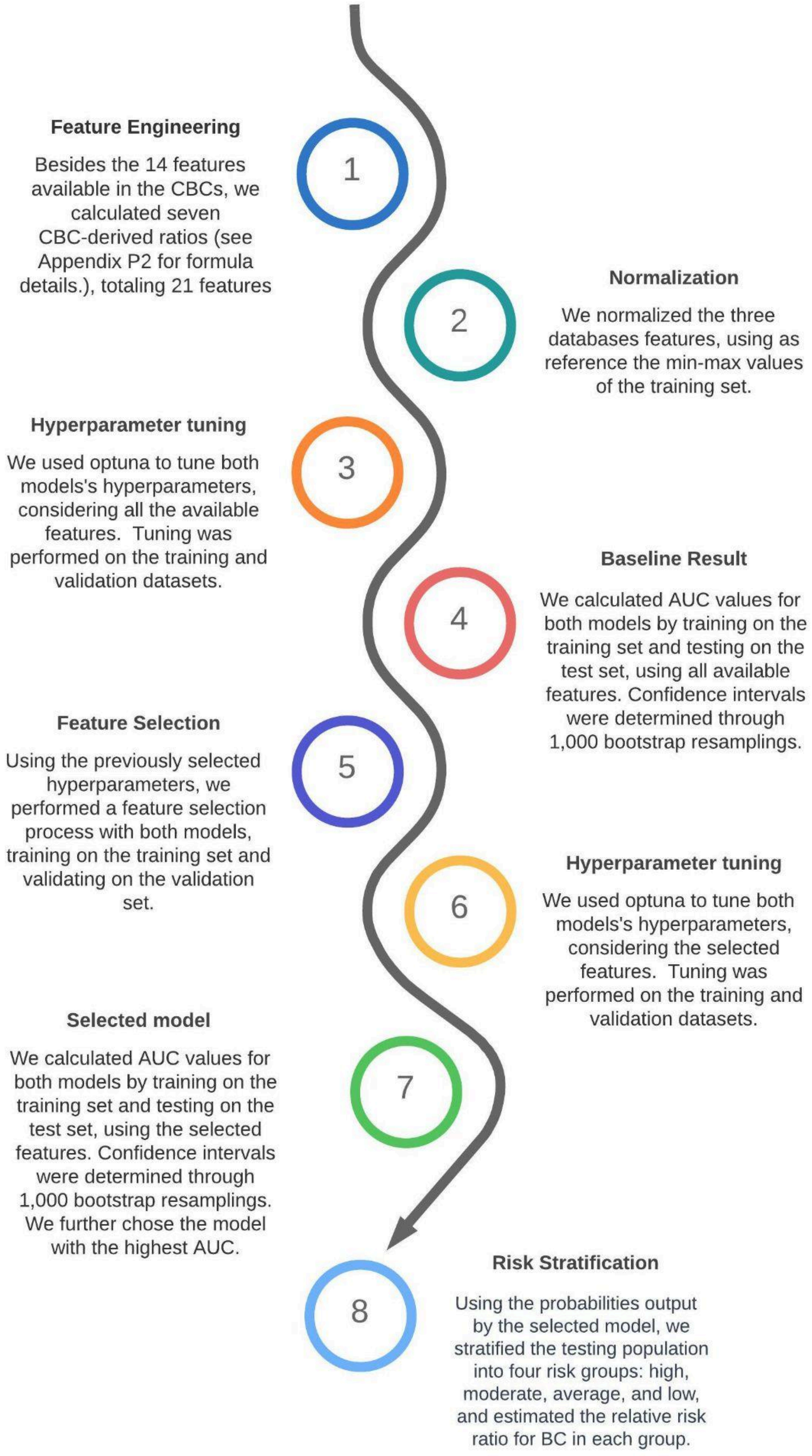

Appendix P4 - Diagram summarizing the methodology steps we have followed in this work.

To assess the risk of breast cancer in our study population, we applied the following direct equation derived from our ridge regression model:

y = $0.0122\, X\, NLR \ +\ 0.003\, X\, Age \ -\ 0.014\, X\, RBC$

where:

NLR is the ratio between Neutrophils and Lymphocytes - both markers are measured in $/mm^3$ Age is measured in years

RBC is the red blood count, measured in $10^6/mm^3$

Based on the value of y, individuals are classified into different risk categories as follows:

If y > 0.15 → high risk

Elif y > 0.12 → moderate risk

Elif y > 0.09 → typical risk

Else → low risk

Appendix P5 - Ridge Model Equation (without normalization) and Thresholds for Risk Classification

Priority Group	1 (High)	2 (Moderate)	3 (Average)	4 (Low)
% of the population	10.0%	20.0%	40.0%	30.0%
% of cancers	19.8%	26.5%	41.1%	12.6%
RR	1.99	1.32	1.02	0.42

Appendix P6 - Average relative risk (RR) for each group.
